# Cerebrospinal Fluid Neurofilament Light Chain Is Associated with Kynurenine Pathway Metabolite Changes in Multiple Sclerosis

**DOI:** 10.3390/ijms21082665

**Published:** 2020-04-11

**Authors:** Cecilia Rajda, Zsolt Galla, Helga Polyák, Zoltán Maróti, Kristóf Babarczy, Dániel Pukoli, László Vécsei

**Affiliations:** 1 Department of Neurology, Faculty of Medicine, University of Szeged, Semmelweis u. 6., H-6725 Szeged, Hungary; polyak.helga@med.u-szeged.hu (H.P.); babarczy.kristof@med.u-szeged.hu (K.B.); vecsei.laszlo@med.u-szeged.hu (L.V.); 2 Department of Pediatrics, Faculty of Medicine, University of Szeged, Korányi fasor. 14-15., H-6725 Szeged, Hungary; galla.zsolt@med.u-szeged.hu (Z.G.); maroti.zoltan@med.u-szeged.hu (Z.M.); 3 Albert Kenessey Hospital, Neurology Unit and University of Szeged, Faculty of Medicine, Department of Neurology Semmelweis u. 6., H-6725 Szeged, Hungary; pukoli.daniel@med.u-szeged.hu; 4 Department of Neurology and Interdisciplinary Excellence Centre, Faculty of Medicine, MTA-SZTE Neuroscience Research Group, University of Szeged, Semmelweis u. 6., H-6725 Szeged, Hungary

**Keywords:** CSF, neurofilament light, multiple sclerosis, quinolinic acid

## Abstract

Neurofilament light (NFL) has proved to be a good prognostic factor in multiple sclerosis (MS), as its level is proportionally elevated with extended neuraxonal damage. The involvement of the kynurenine pathway in neuroinflammation has been proved. The precursor of this pathway is the essential amino acid tryptophan, which is catabolized 95% towards kynurenine metabolites. Quinolinic acid (QUIN) within the brain is only produced in activated microglia and macrophages, leading to axonal degeneration via the activation of N-Methyl-D-aspartate receptors. Neopterin is a biomarker for inflammation produced by macrophages. The association of these biomarkers has not previously been investigated. Our aim was to assess whether there is an association of the neurodegenerative biomarker NFL with the markers of neuroinflammation, e.g., kynurenine metabolites and neopterin, in the cerebrospinal fluid (CSF). CSF samples of patients with MS (pwMS; *n* = 37) and age-matched controls (*n* = 22) were compared for NFL levels by ELISA, while the kynurenine pathway metabolites tryptophan and neopterin were detected with mass spectrometry. Spearman’s correlation showed that NFL is an independent predictor of neurological disability in the MS group. Significant correlations were found between NFL, neopterin, and QUIN, and between kynurenine and neopterin. Receiver operating characteristic (ROC) curve analysis was used to plot the top three best predictors of MS-related disability that yielded the best specificity and sensitivity. Normalized NFL (AUC: 0.923), QUIN (AUC: 0.803), and neopterin (AUC: 0.843) were the best independent predictors of neurological disability in pwMS. The CSF NFL and CSF QUIN, together with neopterin, were elevated in the CSF of pwMS compared to controls. The combination of the neurodegenerative biomarkers together with biomarkers of neuroinflammation could provide additional information on the underlying pathomechanism of disease activity, which is essential for the identification of patients at risk of developing cumulative disabilities.

## 1. Introduction

Neurofilament light chains (NFLs), which belong to the 68 kDa-sized members of the family of filament proteins [1], form the cytoskeleton of neurons. The protein serves as a good prognostic biomarker for different neurodegenerative disorders including multiple sclerosis (MS) [2], and shows an age-dependent increase in both the cerebrospinal fluid (CSF) and the serum with good correlation [3,4]. Thus, tissue injury and regeneration are not restricted to MS; neurofilaments are not specific for the disease, but they can give a picture of its activity and severity [5]. 

**Figure 1:**
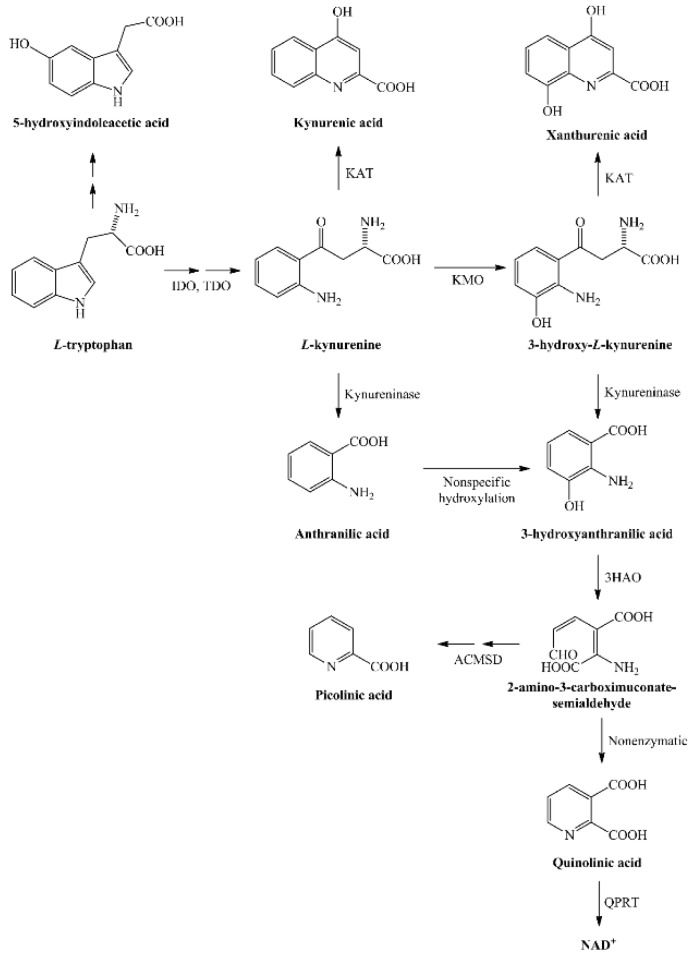
Kynurenine metabolism from its precursor tryptophan to nicotinamide adenine dinucleotide (NAD+) production. Abbreviations: 3HAO—3-hydroxyanthranilate oxygenase, ACMSD—2-amino-3-carboxymuconate semialdehyde decarboxylase, IDO—indoleamine-2,3-dioxygenase, KAT—kynurenine aminotransferase, KMO—kynurenine-3-monooxygenase, NAD+—nicotinamide adenine dinucleotide, QPRT—quinolinic acid phosphoribosiltransferase, TDO—tryptophan-2,3-dioxygenase.

The kynurenine pathway leads to nicotinamide adenine dinucleotide (NAD+) production by degradation of the essential amino acid tryptophan, which is a precursor for serotonin and melatonin as well (Figure 1). Tryptophan is catabolized 95% towards kynurenine metabolites by the rate limiting enzyme *indoleamine-2,3-dioxygenase* (IDO). Its metabolite, quinolinic acid (QUIN), is produced by activated microglia and resident macrophages in the central nervous system (CNS), but not by neurons and astrocytes. QUIN is involved in neuronal death; it acts as an agonist of N-methyl-D-aspartate (NMDA) receptors [6,7] and directly increases glutamate transmission by inhibiting astrocyte glutamate removal and stimulating glutamate release from neurons [8,9]. It acts as a neurotoxic agent by causing blood–brain barrier breakdown, and leads to oxidative stress by inducing lipid peroxidation and mitochondrial dysfunction [10,11] in experimental animal models. It can also induce apoptosis of myelin-producing oligodendrocytes [12]. Neopterin is a nonspecific marker of inflammation present during viral as well as immunological inflammatory processes. Interferon-gamma is capable of increasing neopterin production in macrophages [13,14].

We analyzed data from patients with MS (pwMS) regarding NFL levels in the CSF at diagnostic lumbar puncture (LP) and compared them with tryptophan, kynurenine pathway metabolites, and neopterin in the CSF, retrospectively. As extensive research supported that NFL proved to be a stable, reliable biomarker in MS, especially in the CSF for axonal damage, it served as a proven biomarker for comparison. We hypothesized that elevated quinolinic acid and decreased kynurenic acid levels in the MS group would reflect the pathological processes. A positive correlation of these biomarkers involved in neurodegeneration (NFL) and neuroinflammation (QUIN and neopterin) was expected in pwMS when compared to the control group. 

## 2. Results

### 2.1. NFL Levels Were Measured with ELISA 

We measured the NFL level in 37 CSF samples of pwMS and 22 CSF samples of controls (demographic data shown in Table 1). Significantly higher NFL levels were found in the CSF of pwMS compared to controls (Mann–Whitney U test, *p* < 0.0001, median ± SEM 3280.36 ± pg/mL vs. 572.50 ± 70.23 pg/mL). As linear regression with adjusted R-squared values showed age-dependent changes with regard to NFL (*p* = 0.016, R = 0.545, R-square = 0.297, adjusted R-square = 0.256), we corrected for this effect. The age-corrected NFL values for pwMS were 3021.65 ± 410.13 pg/mL vs. 547.19 ± 66.36 pg/mL (mean ± SD) for controls.

**Figure 2:**
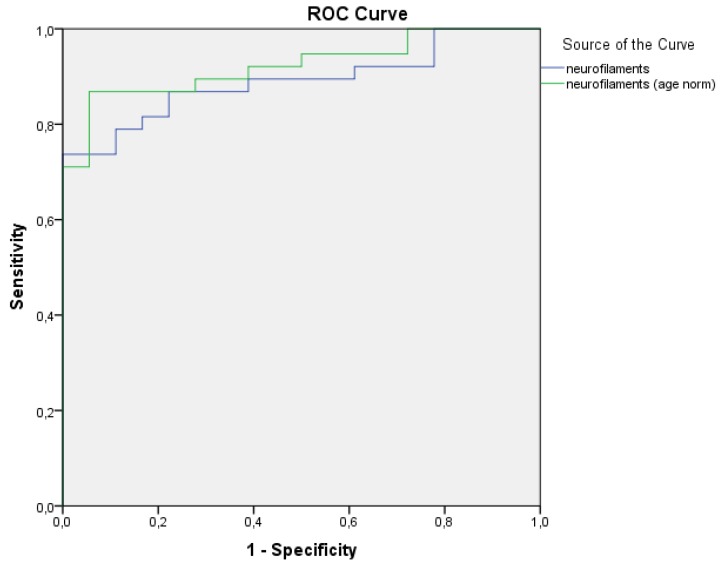
Normalization of the neurofilament light chains (NFL) values yielded higher specificity and sensitivity.

The normalized NFL values gave better specificity and sensitivity (uncorrected for age area under the curve (AUC) AUC_NFL_ = 0.890 and corrected for age AUC_NFLc_ = 0.923, respectively, Figure 2).

### 2.2. Tryptophan, Kynurenine Pathway Metabolites, and Neopterin Were Measured with Mass Spectrometry

**Figure 3:**
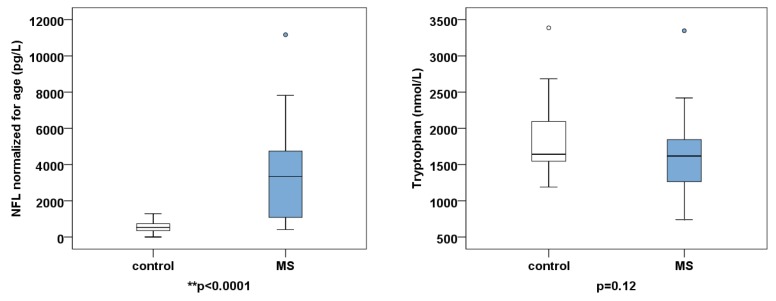

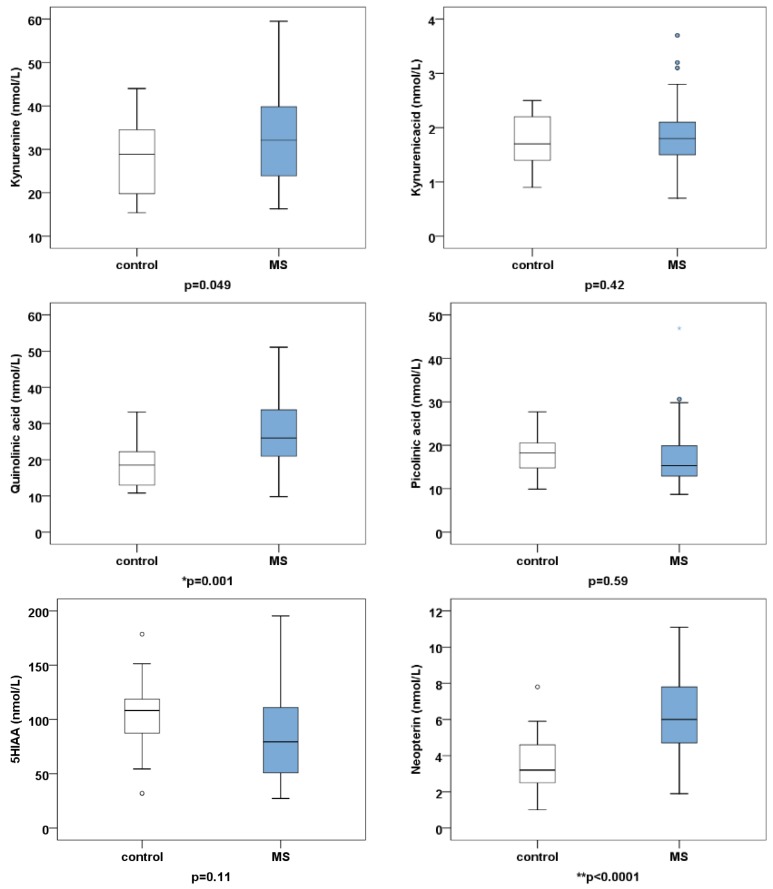
Boxplots of neurofilament light chain (NFL), tryptophan, kynurenine pathway metabolites, and neopterin. The values are given as mean ± SD.* *p* < 0.00625, ** *p* < 0.0001. Abbreviations: 5HIAA—5-hydroxy-indolacetic acid, MS—multiple sclerosis.

The CSF values of tryptophan, kynurenine metabolites, and neopterin of the 37 pwMS and 22 controls are highlighted in Table 2 and Figure 3. The values are expressed as the mean ± SD. To evaluate the significance of the differences between the metabolite levels of disease and control groups, we performed two-tailed independent *t*-tests. For multiple comparisons, the *p* value was considered significant if the *p* value was less than 0.00625 (*p* < 0.05/8).

### 2.3. Correlations between NFL, Tryptophan, Kynurenine Pathway Metabolites, and Neopterin

**Figure 4:**
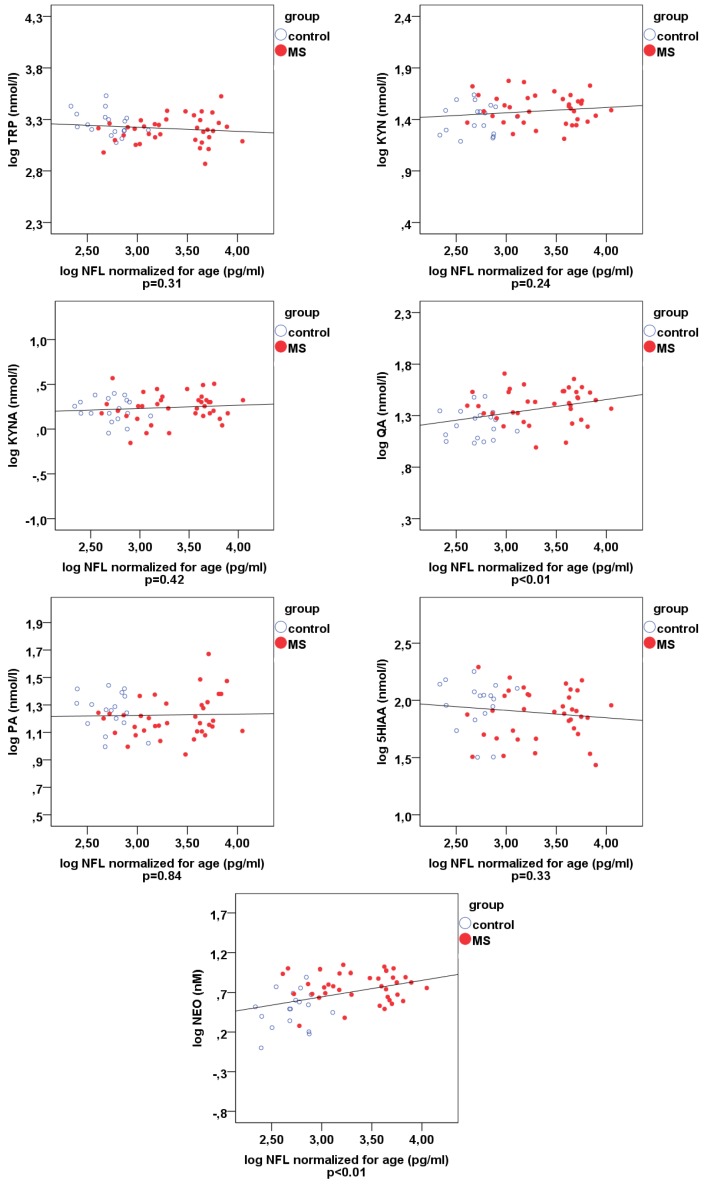
Scatterplots of the Spearman’s rho correlations comparing pwMS and controls regarding NFL, kynurenine metabolites and NEO. *p < 0.00625, ** *p* < 0.000125. Blue ring-plots: controls, red plots: pwMS. Abbreviations: 5HIAA—5-hydroxy-indolacetic acid, KYNA—kynurenic acid, KYN—kynurenine; NEO—neopterin, NFL—neurofilament light chain, PA—picolinic acid, QUIN— quinolinic acid, TRP—tryptophan.

NFL proved to be an independent parameter in the MS group. The correlations between the different metabolites and NFL are shown in Table 3 and in scatterplots in Figure 4. Correlations of 0.01 level significance were found between NFL and QUIN, NFL and neopterin (NEO), kynurenine (KYN) and NEO, QUIN and NEO, KYN and QUIN, and kynurenic acid (KYNA) and 5-hydroxy-indolacetic acid (5HIAA). Correlations of 0.05 significance level were detected between KYNA and QUIN, and between tryptophan (TRP) and QUIN. The correlations between KYNA and 5HIAA, KYN and QUIN, KYNA and QUIN, as well as between TRP and QUIN, are most probably due to the downstream metabolism, which explains these connections. More interestingly, there was a strong positive correlation between NFL, QUIN and NEO, and between KYN and NEO, which points to a positive correlation between the biomarkers of neuroinflammation and axonal degeneration. The only negative correlation was found between TRP and QUIN, which most probably indicates an increased formation of QUIN from the precursor, TRP.

A receiver operating characteristic (ROC) curve was used to plot the true positive rate vs. the false positive rate of the different measured parameters. It depicts the cumulative distribution function of the detection probability on the y-axis and the cumulative distribution function of the false-alarm probability on the x-axis. 

**Figure 5:**
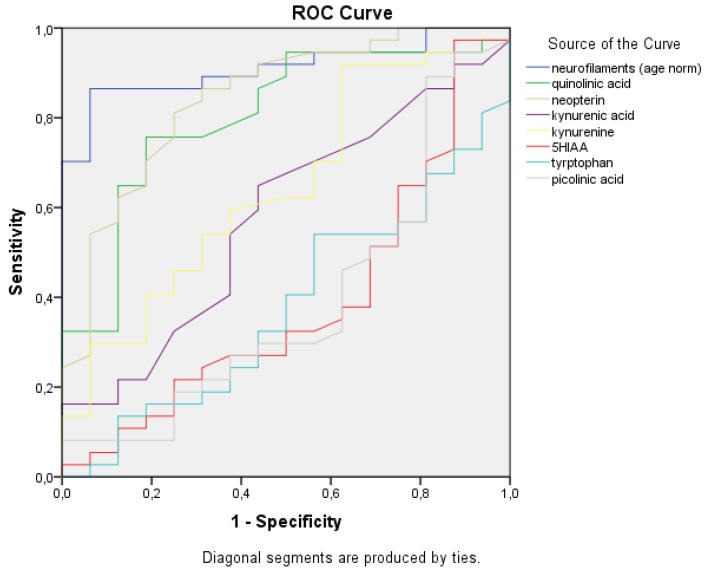
Receiver operating characteristic (ROC) curves of the measured metabolites in the CSF. The ROC curves of the age-normalized NFL, tryptophan pathway metabolites, and neopterin are depicted in different colors. NFL, quinolinic acid, and neopterin showed the best sensitivity and specificity for MS disease activity.

**Figure 6:**
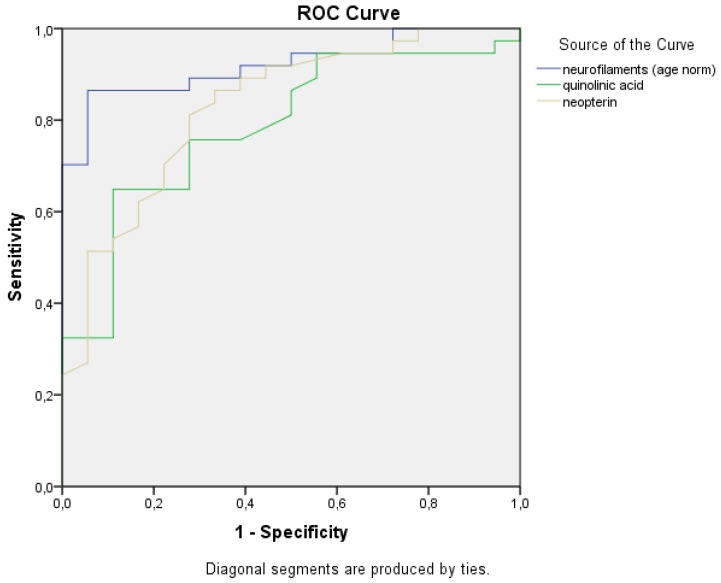
ROC curves for corrected NFLs together with QUIN and neopterin. ROC curves of the best fitting parameters yielding high sensitivity and specificity: NFL, quinolinic acid, and neopterin.

Normalized NFL (AUC: 0.923), QUIN (AUC: 0.803), and neopterin (AUC: 0.843) were the top three best predictors of MS-related disability according to ROC curve analysis among the eight observed values (Figure 5 and Figure 6).

The expanded disability status scale (EDSS) point at the time of LP correlated well with tryptophan, kynurenic acid, and QUIN levels (ANOVA; *p* < 0.001): for tryptophan *r*^2^ = 0.128; y = 9.42*10^16^-0.57 * x; for kynurenic acid *r*^2^ = 0.128, *y* = 9.423*10^16^-0.57 * x; and for QUIN *r*^2^ = 0. 405, *y* =−1.23*10^-15^ + 0.09 * x.

## 3. Discussion

NFL seems to be a robust marker of ongoing disease activity in pwMS. Moreover, NFL levels correlate well with conversion to clinically definitive MS [15,16], increased number of relapses, confirmed disability worsening, treatment and MRI outcomes [17], spinal cord and brain atrophy [4,18,19], and different clinical forms such as radiologically isolated syndrome [20], clinically isolated syndrome (CIS) ([21], relapsing-remitting MS (RRMS) [16,22,23], and primary progressive MS (PPMS) [24]. NFL levels correlate significantly with the presence of gadolinium enchancing lesions and T2 lesion load [20]. Independent of treatment, high NFL levels before treatment are the strongest predicting factor of relapses, accelerated brain volume loss and MRI activity at treatment effect evaluation 24 months after treatment [22]. Several treatments decrease the NFL levels such as natalizumab [25], fingolimod [26,27], and interferon-beta [22]. Gender differences regarding NFL levels have not yet been reported in the literature.

At lower concentrations, QUIN disturbs neuronal homeostasis at the cellular level by phosphorylating the neurofilament subunits [28], while high concentrations induce apoptotic cell death in striatal neurons in cell culture [29,30]. Cocultured astrocytes and neurons interact to protect themselves against QUIN damage [31]. In addition, these neuronal interactions preserve cell morphology and cytoskeletal structure [31]. Furthermore, the neuronal cytoskeleton was found to be more responsive than the cytoskeleton of the astrocytes to QUIN damage, since a ten times higher concentration was needed for cytoskeleton injury in astrocytes than in neurons [30,31]. The morphological changes induced by QUIN interfere with axonal transport and with other axonal cytoskeleton functions [29]. High QUIN levels are associated with neuronal damage in neurodegenerative conditions [28,32]. Furthermore, QUIN treatment led to the disruption of the cytoskeletal homeostasis of neurons, and excitotoxic dendritic damage could be shown [33]. The initial locations of these injuries were in the dendrites [34], and this resulted in apoptosis or necrosis of the neuron, depending on the extent of the damage [35]. In human studies, QUIN levels were increased in both the serum and CSF of pwMS [36]. Lim and coworkers found that QUIN can differentiate MS subtypes [37]. It is proposed that the modulation of the kynurenine pathway could decrease neuroinflammation due to the inhibition of the IDO enzyme and the kynurenine monooxygenase (KMO) enzymes. These proposals are based on previous findings that KMO inhibitors decrease MS disease activity in mouse experimental allergic encephalomyelitis (EAE). In kynurenine-treated mice, a delay in relapse was observed. Kynurenine pathway activation results in two opposite events; the short-term benefits arise from decreased T cell proliferation, leading to immunosuppression, while chronic activation of the kynurenine pathway enzymes enhances production of neurotoxic metabolites and plays a role in preventing the innate repair mechanism of remyelination. In MS, increased QUIN was associated with the loss of oligodendrocytes, astrocytes, and neurons, and decreased neuroprotective metabolites such as kynurenic acid and picolinic acid [38].

Rejdak and coworkers found higher CSF levels of KYNA during relapse and lower levels during remission in relapsing pwMS [39,40]. Interestingly, we have not found changes in CSF KYNA levels, similarly to Aeinehband and coworkers [36]. After stratification based upon the different disease subgroups, relapsing pwMS had a metabolic shift of the kynurenine pathway towards the neurotoxic path (elevated QUIN), while progressing pwMS displayed a trend toward downregulation of the kynurenic pathway. Secondary and primary progressive pwMS differed in KYNA levels, which was much lower in the former group of patients [36].

In MS, besides tissue damage due to relapse, progression independent of relapse activity (PIRA) leads to ongoing accumulation of disability in progressive as well as in relapsing forms of the disease. This chronic condition is characterized by ongoing brain tissue damage, also regarded as faster aging [41,42,43]. Other pathological changes are mitochondrial dysfunction, oxidative stress, and slowly enlarging lesions (SELs) detected on the MRI. SELs may reflect chronic inflammation caused by microglia or macrophage populations resident in the CNS, or they can be present due to secondary Wallerian degeneration [44]. Activated microglia-derived macrophages produce more QUIN than activated microglia, while CSF resident cells do not possess all the enzymes of the kynurenine pathway and are therefore unable to synthesize great amounts of QUIN; however, they can still produce KYN. Astrocytes in the CNS in turn can release extra KYN, which serves as a source for QUIN by monocytes and microglia-derived macrophages. The high levels of QUIN produced by monocytes and these macrophages are toxic to neurons and oligodendrocytes [45]. These features can lead to demyelination and parallelly occurring axonal damage in MS. Further studies are needed to depict the ongoing disease activity in order to allow for the identification of patients at risk of developing cumulative disabilities.

Just as in a recent study in which association between serum NFL and QUIN levels was found in preclinical Alzheimer’s disease [46], we found a strong positive correlation between NFL normalized for age, neopterine, and QUIN, in the CSF of pwMS. This is the first study in which neurodegeneration and neuroinflammation showed a tight correlation in the CSF of MS patients involving independent parameters such as NFL for axonal degeneration, neopterin as a general marker for inflammation, and QUIN as a potential biomarker for neuroinflammation. The limits of the present study are the low subject numbers and that the data are limited to the CSF, which requires an invasive method to obtain biomarkers.

## 4. Methods

### 4.1. Standard Protocol Approvals, Registrations, and Patient Consents 

This study was approved by the local ethical committee (No. 143/25.06.2015). All patients participated voluntarily and gave their informed consent at the admission to the hospital for carrying out medical examinations, for collecting sera and CSF samples for our biobank at lumbar puncture (LP), or later during follow-up. The samples were processed and analyzed according to the international standardized biobanking consensus protocol of the BIOMS-Eu network [47].

### 4.2. Patients and Controls 

Inclusion criteria for MS patients were age > 18 years, LP at diagnosis, at least a 12-month follow-up, and available samples in the biobank obtained between 2012 and 2018. The patients were prospectively followed up, meeting an MS specialist at least four times a year. The diagnosis was based on the revised McDonald criteria [48], which accepted the oligoclonal bands as a proof for dissemination in time, allowing an early diagnosis and treatment. At the consultations, EDSS scores, disease activity, clinical forms, and treatment were assessed regularly. All patients underwent LP during the diagnostic procedure with parallel CSF and blood sampling. None of the patients received MS treatment at the time of LP.

To rule out age-related differences, the CSF samples of pwMS (*n* = 37) were compared with age and gender-matched controls (*n* = 22) with symptomatic neurological disorders (*n* = 12; 8 headache, 2 anxiety, 1 seizure, 1 low back pain) and patients with non-inflammatory neurological diseases (NIND, *n* = 10; 4 lacunar stroke, 4 benign intracranial hypertension, 1 transient global amnesia, and 1 mild ocular myasthenia gravis), and followed for at least 2 years to exclude other underlying pathologies. Patients with known diseases of predictably high NFL levels were excluded (e.g., severe stroke, NMDA-receptor encephalitis).

### 4.3. NFL Analysis 

CSF NFL levels were measured with a commercially available sensitive sandwich ELISA kit (NFL-light ELISA kit; UmanDiagnostics, Umeå, Sweden) using a Multiskan ^®^ EX ELISA scanner (Thermo Electron Corporation, Finland, precision CV < 0.5% (0.3–1.5 Abs) at 405 nm; CV < 1% (1.5–2 Abs) at 405 nm; accuracy ± 2.0% or ± 0.007 Abs whichever is greater, typical value ± 1% (0–2.0 Abs) at 405 nm. The detection limit of this method is given as the limit of quantification of the assay, which was 310 pg/mL. The inter and intraassay variations were below 10%.

### 4.4. Tryptophan, Neopterin, and Kynurenine Pathway Metabolite Analysis 

Parallel to the NFL analysis, CSF samples were measured for kynurenine pathway metabolites.

All reagents and metabolites were analytical reagent grade and were purchased from Sigma-Aldrich or Merck. Deuterated internal standards were purchased from Cambridge Isotope Laboratories and Medical Isotopes Inc. Concurrent analysis of tryptophan, kynurenine, quinolinic acid, kynurenic acid, picolinic acid, 5-hydroxyindoleacetic acid, and neopterin was performed with LC-MS/MS as described by Fuertig et al. [49], using an injection volume of 15 μL of the prepared extract from each samples. The LC-MS/MS system consisted of a PerkinElmer Flexar UHPLC system (two FX-10 binary pumps, solvent manager, autosampler, and thermostatic oven; all PerkinElmer Inc.) coupled with an AB SCIEX QTRAP 5500 MS/MS triple quadrupole mass spectrometer and controlled by the Analyst 1.6.2 software (both AB Sciex, Framingham, MA USA). MS-analyses were performed in positive electrospray ionization mode (ESI); interface parameters were set as follows: curtain gas 40 psi; ionspray voltage 5500, probe temperature 700 °C; nebulizer gas 40 psi; heather gas 40 psi. The quadrupoles operated in multiple reaction monitoring mode. Dwell time was set to 20 msec of each transition, using nitrogen as collision gas.

### 4.5. Statistical Analysis

Statistical calculations were performed with the IBM SPSS Statistics 24 software (IBM Corp., Armonk, NY, USA).

Except in the case of NFL, all measured parameters had normal distribution in both patients and controls. Therefore, Mann–Whitney U test was performed for NFL, and the data were presented in median ± SEM. For the other parameters independent sample t-test was used, and the values were given in mean ± SD. NFL proved to be age dependent by linear regression (*p* = 0.016; r2 linear = 0.297, *y* = 1.28*10^2^ + 15.77 * x) and served as a basis for normalization for further use.

We performed Spearman rank correlation to analyze the relations between the measured metabolites.

A receiver operating characteristic (ROC) curve was used to plot the true positive rate vs. the false positive rate of the different measured parameters.

## 5. Conclusions

The recent diagnostic criteria of MS [45] enable the early stage diagnosis of this potentially disabling disease beginning mainly in young adulthood, thus also facilitating early treatment. In MS, disability accumulates not only when relapse occurs but also between relapses, pointing to a continuously ongoing neurodegeneration besides neuroinflammation. Early, appropriately effective treatment can hinder the accumulation of disability. We found that CSF NFL and CSF QUIN, together with neopterin, were elevated in the CSF of pwMS compared to controls. Combined measurement of the neurodegenerative biomarkers together with biomarkers of neuroinflammation could provide additional information on the underlying pathomechanism of disease activity, which is essential to identify patients at risk of developing cumulative disabilities.

Reliable biomarkers are needed which could additionally direct attention towards patients who need highly effective disease-modifying treatment at the early stages of the disease.

## Figures and Tables

**Table 1:** Demographics of the patients and controls.

	pwMS	Controls
No.	37	22
No. male:female	18:19	11:11
median age (year±SEM)	34.1 ± 9.9	38.6 ± 10.6
clinical course (2017 diagnostic criteria)	CIS *n* = 5 (*n* = 2 converted later to RMS) RMS *n* = 32	symptomatic *n* = 12 NIND *n* = 10
EDSS at onset (median±SEM)	2.0 ± 1.4	
EDSS at FUP (median±SEM)	1.0 ± 2.3	

CIS—clinically isolated syndrome, EDSS—expanded disability status scale, FUP—follow up, RMS—relapsing multiple sclerosis, SEM—standard error of mean, pwMS—patients with multiple sclerosis.

**Table 2:** Cerebrospinal fluid (CSF) values of tryptophan, kynurenine pathway metabolites, and neopterin.

Group	pwMS (*n* = 37)	Controls (*n* = 22)	*p* Value
tryptophan (nmol/L)	1642.57 ± 510.96	1864.17 ± 530.83	*p* = 0.12
kynurenine (nmol/L)	33.58 ± 11.07	27.99 ± 8.88	*p* = 0.049
kynurenic acid (nmol/L)	1.87 ± 0.66	1.73 ± 0.48	*p* = 0.42
QUIN (nmol/L)	26.78 ± 9.38	18.69 ± 6.59	* *p* = 0.001
piconilic acid (nmol/L)	17.36 ± 7.16	18.31 ± 5.16	*p* = 0.59
5HIAA (nmol/L)	83.99 ± 39.95	100.97 ± 36.45	*p* = 0.11
neopterin (nmol/L)	6.29 ± 2.40	3.51 ± 1.61	** *p* < 0.0001

The values are given as mean ± SD. * *p* < 0.00625, ** *p* < 0.000125. Abbreviations: QUIN—quinolinic acid, 5HIAA—5-hydroxy-indolacetic acid.

**Table 3:** Correlation between NFL, tryptophan, kynurenine pathway metabolites, and neopterin levels.

Spearman’s rho Correlation Coefficient	NFL	TRP	KYN	KYNA	QUIN	PA	5HIAA	NEO
NFL	1	−0.118	0.165	0.095	0.366 **	−0.016	−0.139	0.364 **
TRP	−0.118	1	0.082	−0.125	−0.313 *	0.081	0.113	−0.225
KYN	0.165	0.082	1	0.251	0.508 **	−0.203	0.026	0.386 **
KYNA	0.095	−0.125	0.251	1	0.327 *	0.030	0.576 **	0.067
QUIN	0.366 **	−0.313 *	0.508 **	0.327*	1	−0.121	−0.017	0.513**
PA	−0.016	0.081	−0.203	0.030	−0.121	1	−0.018	−0.074
5HIAA	−0.139	0.113	0.026	0.576 **	−0.017	−0.018	1	−0.217
NEO	0.364 **	−0.225	0.386 **	0.067	0.513 **	−0.074	−0.217	1

* Correlation is significant at the 0.05 level (2 tailed), ** correlation is significant at the 0.01 level (2 tailed). 5HIAA—5-hydroxy-indolacetic acid, KYNA—kynurenic acid, KYN—kynurenine, NEO—neopterin, NFL—neurofilament light chain, PA—picolinic acid, QUIN—quinolinic acid, TRP—tryptophan.
